# Straw use and availability for second generation biofuels in England

**DOI:** 10.1016/j.biombioe.2013.02.033

**Published:** 2013-08

**Authors:** Neryssa J. Glithero, Paul Wilson, Stephen J. Ramsden

**Affiliations:** Division of Agricultural and Environmental Sciences, School of Biosciences, University of Nottingham, Sutton Bonington Campus, Loughborough LE12 5RD, UK

**Keywords:** Cereal straw, Bioenergy, Second generation biofuels, Feedstock availability, Farm Business Survey

## Abstract

Meeting EU targets for renewable transport fuels by 2020 will necessitate a large increase in bioenergy feedstocks. Although deployment of first generation biofuels has been the major response to meeting these targets they are subject to wide debate on their sustainability leading to the development of second generation technologies which use lignocellulosic feedstocks. Second generation biofuel can be subdivided into those from dedicated bioenergy crops (DESGB), e.g. miscanthus, or those from co-products (CPSGB) such as cereal straw. Potential supply of cereal straw as a feedstock for CPSGB's is uncertain in England due to the difficulty in obtaining data and the uncertainty in current estimates. An on-farm survey of 249 farms (Cereal, General Cropping and Mixed) in England was performed and linked with Farm Business Survey data to estimate current straw use and potential straw availability. No significant correlations between harvested grain and straw yields were found for wheat and oilseed rape and only a weak correlation was observed for barley. In England there is a potential cereal straw supply of 5.27 Mt from arable farm types; 3.82 Mt are currently used and 1.45 Mt currently chopped and incorporated. If currently chopped and incorporated cereal straw from arable farm types was converted into bioethanol, this could represent 1.5% of the UK petrol consumption by energy equivalence. The variations in regional straw yields (t ha^−1^) have a great effect on the England supply of straw and the potential amount of bioethanol that can be produced.

## Highlights

► On-farm survey of English arable farms to determine cereal straw use and availability. ► Estimated 5.27 Mt of cereal straw from arable farms, of which 27.5% is incorporated. ► Incorporated cereal straw on arable farms could supply 1.5% of UK petrol consumption. ► Crop residue co-products offer potential as sustainable biofuel feedstock. ► East Midlands and East of England provide potential English biofuel feedstock supply.

## 1 Introduction

Concerns about energy security and the environmental impacts of energy production have led to the implementation of policies designed to encourage the production and use of renewable bioenergy [1], [2], [3]. By 2020 EU legislation requires that 20% of energy must be produced from renewable sources, with 10% of transport energy (final use) derived from a renewable source (EU, Directive 2009/28/EU) [4]. The USA has similar legislation in place that calls for 36 billion US gallons of renewable liquid fuel to be used by 2022 (USA, Public Law 110-140 (2007)). At present ‘first generation’ bioenergy has been the major technology deployed to meet these renewable targets [5], [6], particularly in the transport fuel sector. However, first generation biofuels ferment starches and sugars from food crops (e.g. sugar beet/cane, corn and cereal grains) into liquid biofuels, leading to widespread concern over competition with food production; this aspect being further highlighted during times of increased food prices and food shortages [7], [8], [9], [10]. In response, second generation technologies are being developed, often through public-private research initiatives (e.g. the UK's BBRSC Sustainable Bioenergy Centre [11]; the EBI [12] programme in the USA) which use lignocellulosic feedstocks (e.g. miscanthus, cereal straw). Glithero et al. (2012) [13], further sub divide second generation biofuel into dedicated energy crop second generation biofuel (DESGB) and co-product second generation biofuel (CPSGB), with the latter utilising co-products from ‘food’ crops (e.g. cereal straw; corn stover). Institutional and private interest in second generation technologies includes mandatory inclusion of lignocellulosic biofuel in the USA, and private sector investment in processing facilities by Inbicon in Denmark [14], [15]. Within the UK, dedicated energy crop production remains an ‘infant industry’, with miscanthus and short rotation coppice currently only accounting for 0.044% of agricultural land use [16].

Dedicated energy crops offer the potential for efficient energy production per hectare – for example, some studies show that they are less demanding in their use of inputs than food crops [17] thus providing energy input savings per hectare; however, the overall energy efficiency of biofuel produced from wood or miscanthus (switchgrass) biomass sources is significantly lower than from biofuel produced directly from grains [18]. Moreover, farmer uptake of dedicated energy crops is anticipated to remain low in the foreseeable future [19], [20]. By contrast, large areas of cereal crops are grown in the UK (3.013 million hectares of wheat, barley and oats [21] accounting for 16.5% of agricultural land use), with production being greatest in the eastern parts of the country. Production data on the area of cereal crops grown is known, together with industry estimates of quantities of grain produced. However, data on the quantities of straw produced, and the current utilisation of cereal straw (e.g. use in animal bedding/feed, in-field protection of high value crops, co-fired energy production, incorporation into the soil), is lacking. Estimates from a Defra-funded FBS (Farm Business Survey) survey of energy use on English farms in 2007 suggest that soil incorporation of straw occurs on 50% of cereal area (authors' calculations). Based on crop data, grain to total biomass yields, harvestable straw yield data and requirements for straw by other agricultural sectors, Copeland and Turley (2008) [22], estimated there was a ’surplus‘ of 5.7 million tonnes of straw, from all crop types, in Great Britain. However, in practise, the availability of cereal straw for a bioenergy plant depends on numerous factors, including biomass produced within the field, harvesting height of the straw, cereal varieties grown and the relative proportions of straw to grain biomass. On arable farms in particular, direct straw incorporation into the soil is often practised, potentially providing soil organic carbon enhancement [23], [24] and soil nutrients [13]; moreover, commercial farming practise is heavily influenced by policy incentives and directives, and soil health represents a key element of current Common Agricultural Policy (CAP) proposals [25]. In Denmark, growing interest in straw as an energy source has led to a more detailed assessment of the availability of straw for bioenergy production [15]; it was concluded that a significant change in Danish straw supply could arise from minor changes in the grain and straw yield relationship at production level.

Given recent research and industry investment, together with favourable regulatory frameworks, second generation biofuels look set to be an important component of the bioenergy mix that is required to meet EU Directive 2009/28/EU [4]. While lignocellulosic processing plants may look to generate both DESGB and CPSGB, the potential for cereal straw to provide large quantities of feedstock without substantially compromising food production currently exists, and offers immediate potential as a feedstock to the sector. However, dedicated energy crops and cereal straw are both low-value bulky products, with transportation costs accounting for a large proportion of the delivered value of feedstocks. This raises two immediate questions with respect to industry logistics; first what is the total quantity of feedstock potential to supply a second generation bioenergy plant? Second, given the financial and energy costs of feedstock transportation, where do large quantities of ‘surplus’ cereal straw exist that are currently being incorporated into the soil? In order to address these questions we undertook a large-scale on-farm survey of arable farmers throughout England who also participated in the FBS. Survey data was linked to crop production and farm business data available from the FBS. The objectives of this paper therefore are to: (i) describe the scope and methodology of the on-farm survey, (ii) present the survey findings in relation to straw use in England, (iii) aggregate survey data to provide estimates for regional and national (English) straw supplies and use, (iv) investigate potential linkages between straw and grain yields in England, and v) consider the geographical implications of these findings in relation to location of a bioenergy processing plant and arable soil health. The survey scope, structure and sampling strategies are given in Section 2, together with the data aggregation and analysis methodologies. Observed straw yields, grain/straw relationships and aggregated straw use and values are then presented in Section 3. The implications of these findings are discussed in detail in Section 4.

## 2 Methods

Previous approaches for quantifying crop residue availability include the use of Geographical Information System (GIS) assessment dependant upon regional crop yields, harvested areas and residue to seed/crop product ratios [26]. For example Monforti et al. [26] built upon and updated previous studies [27], [28] to estimate total UK crop residues of 20.4 Mt dry matter production of which 8.37 Mt were collectable and 4.2 Mt were available. However, wide ranging estimates of residue to seed ratios are frequently observed in these approaches (e.g. 0.6-1.75 for wheat; 0.9-1.8 for barley), which are further confounded by estimates of recoverable residue to seed ratios (0.8-1.6 for wheat; 0.8-1.3 for barley) [27]. Other researchers have used statistical data obtained from agricultural surveys, accounting for supply of and demand (livestock and humus/soil incorporation requirements) for straw in their analyses; based on an analysis of survey data for the Baden-Württemburg state of Germany, approximately 30% of straw was calculated to be ‘surplus’ [29]. Survey information can be potentially revealing, particularly with respect to regional variation. Within the UK, evidence suggests that 22% of cereal straw remains on the field following baling of cereal crops [30], while other estimates indicate that approximately 50% of stubble, chaff and uncollected straw are returned to the soil after baling [31]; again demonstrating the uncertainty surrounding harvested straw yields. On-farm anecdotal evidence also indicates that grain-straw relationships are not robust with respect to harvestable grain and straw yields. This is due to variation in on-farm practices e.g. cereal varietal choice, use of plant growth regulators to shorten straw height, crop harvesting techniques, cutting height, climate and soil conditions [26].

It is therefore argued that robust reliable estimates of grain to straw biomass relationships do not exist in the literature, and moreover, in practical contexts it is the ‘harvested’ grain to straw yield relationship which is of direct interest in contrast to ‘produced’ grain-straw biomass ratios. Moreover, estimates based upon average crop yields, harvested areas and residue to crop ratios do not capture the variation that exists around the average estimates, albeit that both production and harvestable residue to crop ratios are observed to vary considerably [27]. A direct survey approach was therefore taken to obtain data on straw use and volumes on arable farm types (Cereal, General Cropping, Mixed) in England. Production records generally do not exist for this co-product (i.e. unlike grain production, most arable farmers do not accurately measure tonnages of straw produced) [26]. The advantages of the direct survey approach include the ability to specify questions to obtain data not previously captured (e.g. specific uses of straw by crop type) and to obtain individual farm-level observations against which to assess variation in straw yields. Disadvantages of the direct survey approach include the associated cost and the difficulty in accurately capturing yield data on a relatively low value by-product where production estimates must be largely obtained from knowledge held by the farmer rather than specific crop production records (e.g. estimates of bales of straw produced in contrast to cereal grain for which more precise records are more commonly found). The sub-sections below specify the details of the survey approach undertaken including those relating to sample size constraints and representative coverage of the main arable farm types across the regions of England.

### 2.1 Questionnaire design and piloting

The survey questionnaire contained a variety of questions on straw use, straw volumes baled, crop cultivations, cereal variety choice, straw incorporation, contract implications of bioethanol production and dedicated bioenergy crops. Following Oppenheim [32] appropriate questionnaire design techniques (funnelling of questions and including a combination of closed, rating scale and open questions), were adopted during the design phase. Formulation of the questions drew upon expert knowledge of the sector and ‘knowledge transfer’ events with arable farmers (e.g. the UK *Cereals*^1^ event, on-farm farmer-discussions) to inform both questions of interest and detailed options for possible answers. On-farm survey data were obtained by using experienced FBS Researchers Officers (ROs) from Rural Business Research (RBR) units in England as part of the annual FBS research programme (see Section 2.2). It is worth noting that the FBS is part of the European Union's Farm Accountancy Data Network. Prior to full implementation, the questionnaire was piloted by RBR ROs during Dec 2010 to February 2011 and feedback from both farmers and RBR ROs was incorporated into the questionnaire, leading to questions being removed, or reworded for greater clarity, to allow accurate responses to be obtained. The final questionnaire was extensive in coverage and designed to be linked to production and business data from the FBS. Specific questions of relevance to this paper relate to the use and volume of straw removed from arable land (questions presented in Appendix A). These questions covered the use of wheat, barley and oilseed rape straw and the percentage crop area associated with different uses of each type of straw. Potential straw uses were: chopped and incorporated, baled for on-farm livestock use, baled for on-farm crop use (e.g. providing winter protection for carrots), baled and sold for an agricultural use, baled and sold for an industrial use, baled and sold for any other use (e.g. horses, thatching), and sold ‘in swath’. Where straw was baled the number of bales was recorded according to the type of bale: large and small Hesston, large-round, small-square and other^2^. These data related to the 2010 crop harvest straw use. To assess variability in straw use decisions across years, farmers were additionally asked if the planned 2011 harvest straw use would be the same as 2010. Where straw use differed between the two harvest years, the planned 2011 harvest straw use was also recorded. The questions asked, combined with the FBS data on cropping areas for 2010 harvest, allow estimates of straw volumes and uses to be calculated, as detailed in Section 2.3.

### 2.2 Data collection

The FBS is the most authoritative independent data source on farm business practices and performance in England. Utilising the FBS research programme, and drawing upon the expertise of the ROs, ensured that in addition to data collected specifically for the survey, data such as cropping areas, grain yields and location (e.g. Government Office Regions, GOR) were available from FBS data. On-farm interviews took place between February and October 2011. The FBS research programme sample is based upon population data from Defra's annual June Survey returns of the structure of the industry to ensure that the FBS is stratified to reflect population practise by farm type by GOR. The survey was carried out on the main arable farm types within the FBS; Cereals, General Cropping, Mixed. A sub-sample of the FBS sample was set at approximately 46% of farms within these three arable farm types and across three size groupings within these farm types. The three farm arable types of Cereals, General Cropping and Mixed were determined to be those most likely to be interested in supplying straw for off-farm use (e.g. bioenergy production), it being further assumed that other farm types (livestock based) that grow cereal crops would mainly use any straw produced for their own use and will generally not engage in the external market for straw to any great extent; the three main arable farm types covered in the survey therefore capture the potential capacity of English farmers who may engage in a market for straw-feedstock biofuel production. With respect to complete data returns for questions two, three and four of the survey, which form the basis of this analysis, 249 farm observations are available, with the number of observations by farm type and GOR presented in Table 1.

### 2.3 Data analysis

The percentage of land in different uses for straw (e.g. incorporation, baling for agricultural use) for each crop (wheat, barley and oilseed rape) was collected for each farm (categories described in Section 2.1 and Appendix A). These straw use data were converted into straw use area (SUA) data by multiplying the percentages by the area of each crop for each farm. Aggregation of the SUA data by area weighting gives the regional straw use area (RSUA). Following advice from Defra Statisticians (Langton, *pers. com*), the aggregation method is based upon deriving ‘area weights’ from calculating the population areas of wheat, barley and oilseed rape (by farm type and GOR from the June Survey [21]) divided by the survey areas (these are specific to the crop, farm type and GOR). This provides area weighting values by crop, GOR and farm type. Where there were fewer than five farms within a GOR and farm type grouping, data relating to two farm types were combined to ensure that very small sample survey returns were not used to represent all farms of that type within a region. This combining procedure has been carried out for the North East, North West, South East and South West GORs where the General Cropping farms have been combined with the Mixed farms. The RSUA is calculated by multiplying the SUA by the area weights and then aggregating this for the farms in each GOR.

As straw quantity data was obtained from the number of bales produced, straw yields for each farm and crop were calculated on the basis of industry weight estimates for straw bales as presented in Table B1 in Appendix B, multiplied by the number of bales produced and the area of the crop on farm that was baled. Individual farm straw crop specific yields were then analysed as a function of individual farm crop specific grain yields. Estimates where straw yields exceeded grain yields were deemed to represent data entry errors (e.g. incorrect bale type specified) and were therefore excluded from the calculations for straw yields and potential volume calculations. Statistical analysis, in the form of a normality-test (Shapiro–Wilk test), of the included straw yields was performed and further analyses undertaken where appropriate. Regional straw yields (RSYs, t ha^−1^) were calculated based on the total tonnage from all farms in the GOR, and the total area from which this straw value was obtained.

The potential supply of straw at the GOR and England level was calculated from the RSUA and the RSYs. This also provides the supply of straw that is currently used in some form and the straw supply that is currently chopped and incorporated into the soil. From these values the England straw yields (t ha^−1^) were calculated.

The advantages of calculating straw use data using the above approach which aggregates from farm to national levels includes examination of the variation in straw use and per hectare yields that exists across different farm types and different regions, which then further informs policy makers and industrialists as to the potential supply of straw within defined geographical regions, and moreover the variation that may exist between different regions. Given the low density and relatively low value of straw as a product, commercial biofuel production based upon straw feedstock will arguably be regionally dependant. While such analyses could be undertaken on the basis of average straw yields and harvested areas based upon national data [26], [27], [28], differences in on-farm straw use practices and yield variations could not be accounted for using this national accounting procedure; aggregation from farm to national levels using robust aggregation methods permits these variations to be accounted for. A potential disadvantage of aggregation from farm to national level based upon a single year's observation is the year to year variability in straw yield which may occur. We recognise this as a potential limitation to our approach and place our results in the context of the particular growing season within the discussion section.

## 3 Results

### 3.1 Harvested grain to straw yield relationships and England yields

**Fig. 1:**
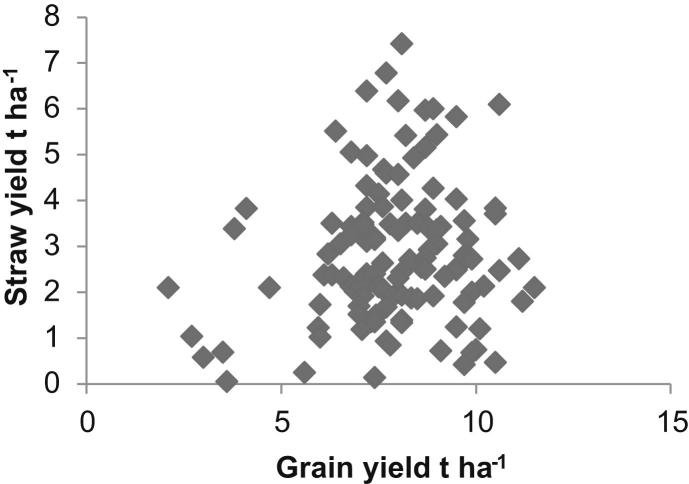
Grain to straw yield relationships for wheat.

**Fig. 2:**
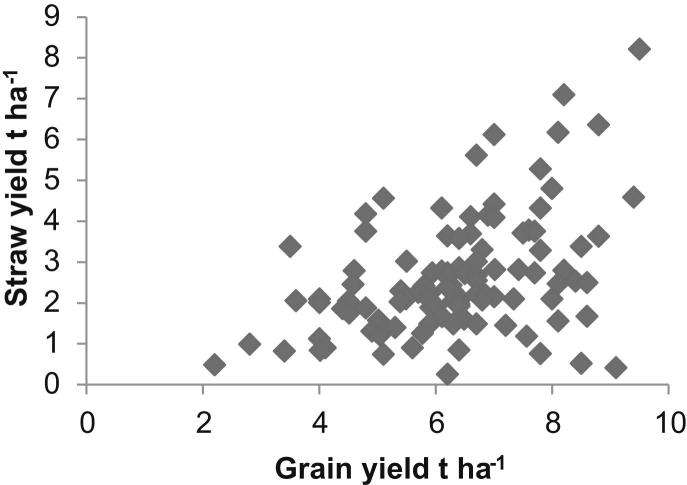
Grain to straw yield relationships for barley.

**Fig. 3:**
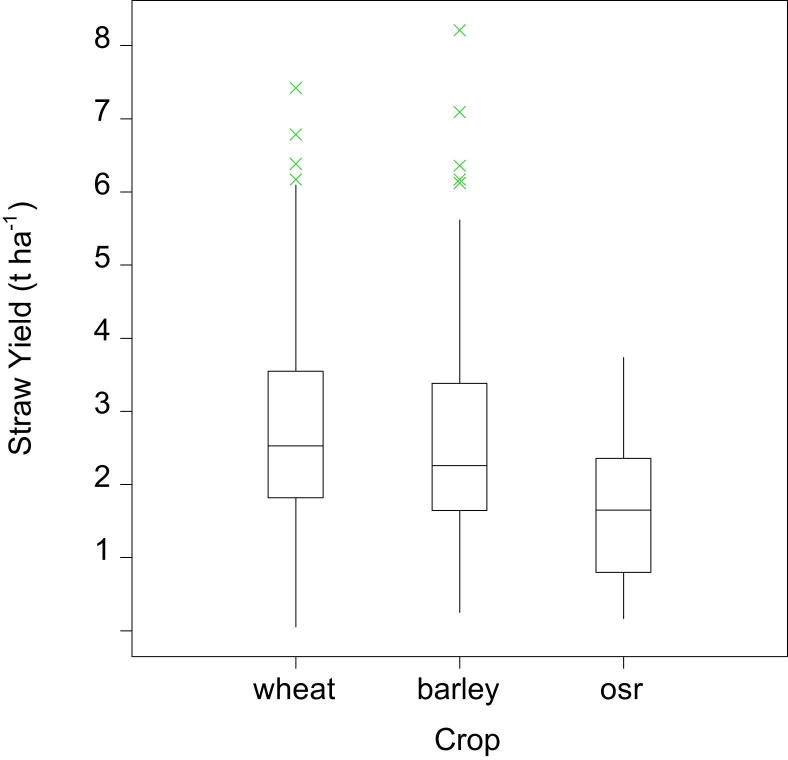
Wheat, barley and oilseed rape (osr) straw yield distribution (wheat *n* = 119, barley *n* = 105,s osr *n* = 9, n is the number of sample points).

From the 249 arable farm observations, 227 farms grew wheat, 162 barley and 140 oilseed rape. Combining the 2010 FBS data with the survey information allows average harvestable straw yields to be calculated and compared to grain yields. There is no clear relationship between harvested grain to straw yields for wheat across England, Fig. 1, as indicated by the Spearman's rank correlation coefficient (SRCC) of 0.126 which is not statistically significant. In addition, there is no statistically significant relationship between harvested grain and straw yields for oilseed rape (SRCC of −0.271). For barley, the SRCC between the harvested grain and straw yields, Fig. 2, is 0.410 (statistically significant at the 99.9% level) showing a weak relationship. The variation in grain to straw yields observed for the three crops is greater than would be expected from previous estimates of residue to grain ratios [27]; however, the estimates provided here are based on individual farm-crop specific data in contrast to the average values observed in the literature. Nevertheless, there are particular observations indicating very low straw yields against relatively high grain yields within the data presented. Potential explanations for these observations include the survey technique adopted: the farmer responder provided the researcher with an estimate of the number of crop-specific straw bales produced from a particular percentage of crop-specific area. Given the relatively low value of straw as an output from arable production, it is plausible that inaccuracies in the farmer's recall of both number of bales produced and the percentage area from which straw was baled could have occurred leading to observed low yields. In addition, the 2010 harvest year included a period of low rainfall, particularly in the East of England from March to July 2010, and straw yields were noted to have been lower than in typical years leading to high market prices for straw [33], [34]. Average English harvestable straw yields for wheat, barley and oilseed rape from arable farms are estimated to be 2.53 t ha^−1^, 2.26 t ha^−1^ and 1.65 t ha^−1^ respectively (median values) for the 2010 harvest. The straw yield data for wheat, barley and oilseed rape, Fig. 3, shows the range of the data for these crops. The median values for wheat and barley have 95% confidence intervals of (2.31, 3.06) and (2.07, 2.67) respectively. The median values reported are more relevant than means due to the non-normal distribution of the straw yield data for these crops (wheat and barley), tested using the Shapiro–Wilk test for normality (wheat (*n* = 119), barley (*n* = 105) both of which are significant at the 99% level of significance indicating that the null hypothesis that the distribution is normal is rejected). Data observations for oilseed rape were limited, hence it was deemed inappropriate to test for normality for this crop.

### 3.2 Cereal area practices

The areas of wheat, barley and oilseed rape on arable farm types by GOR can be seen in Table 2. There are distinct differences between GORs, for example the only substantial area of barley straw incorporation is in the East of England where 51% of the straw from the barley crop area is incorporated, accounting for 98% of all incorporated barley straw area in England; the East of England also records the largest percentage of wheat straw incorporated (64%), equating to 53% of the total wheat straw incorporated area. In aggregate, 36%, 18% and 87% of the straw areas for wheat, barley and oilseed rape on arable farm types respectively are incorporated. Table 2 also shows that only a small percentage of wheat straw is currently baled for industrial use by farmers; no oilseed rape or barley straw is baled for this use on the basis of the survey returns. However, there are substantial areas of wheat and barley straw across England that is ‘sold in swath’, and hence the destination or use of this straw which is baled, removed and marketed by a third party, is unknown and indicates further current agricultural or industrial use of straw being supplied via this third party arrangement. Straw use for 2011 was stated to be the same as the 2010 harvest on 87% of the farms, indicating broad consistency of approach to straw use between the two harvest years.

### 3.3 Regional straw yields

Regional variation in straw yields exists as presented in Table 3, and highlights the importance of understanding variation in both regional straw use practices and regional straw yields. The total tonnage of wheat and barley straw that is incorporated on arable farm types is less than the tonnage used in all GORs with the exception of the East of England. In general, the majority of the barley straw is used in some form. Given that the majority of oilseed rape straw area was incorporated, there were insufficient data observations on baled oilseed rape straw to calculate regional straw yields; hence, only a national yield is presented for this crop.

Overall, the data available suggest that 1.45 million tonnes of harvestable cereal straw is currently incorporated from Cereal, General Cropping and Mixed farms in England although there is considerable variation in harvestable straw yields reported. As noted, the potential for farmer recall with respect to bales produced and crop areas baled may be associated with a lower degree of accuracy than would be observed for grain yield data; the particular cropping year characteristics may also have contributed to low straw yields in 2010 [33], [34]. Assuming that 1.45 Mt is a realistic initial estimate, there is scope for improvement on this figure through varietal choice, crop management, crop harvesting techniques and cutting height [26] – all these factors will vary from farm to farm. From the limited data available for oilseed rape yields there is at least 700,000 tonnes of this straw in England from the three arable farm types that is currently incorporated into the soil, offering further feedstock potential for bioenergy purposes. However, as with wheat and barley, crop residue incorporation may provide benefits with respect to soil organic carbon content and fertility for the following crops [31], [35]; these would be partially lost, the extent depending on the cutting height at harvest. Ranking the results by GORs by per hectare wheat straw yields, from greatest to least (Table 4), reveals no clear East/West or North/South trends.

### 3.4 Straw Usage by GOR

**Fig. 4:**
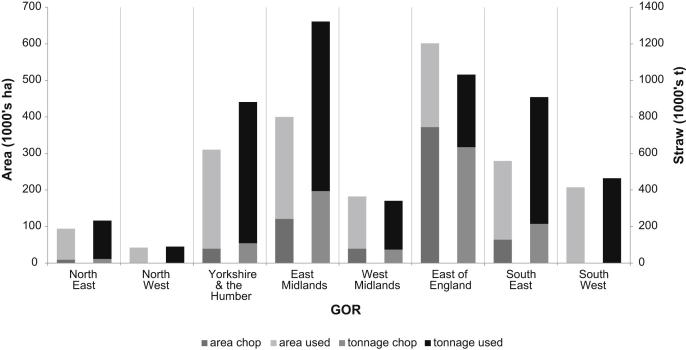
Cereal area and straw use on arable farm types for wheat and barley combined by GOR. Left hand columns relate to the area of wheat and barley in England and the use of straw for that area. The right hand columns relate to the straw supply from wheat and barley combined and the amount that is used or incorporated for each GOR.

Examining wheat and barley straw use on arable farm types by area and tonnage by GOR further highlights the importance of regional straw yield variations (Fig. 4). Note that while the East of England grows the largest area of wheat and barley it does not produce the largest potential tonnage of cereal straw in England. The East Midlands is estimated to produce the largest tonnage of cereal straw, while the East of England contains the greatest tonnage of incorporated cereal straw in England.

## 4 Discussion

The estimated total potential production of 4 Mt of wheat straw from arable farm types in England is lower than the 5.9 Mt quoted by Ref. [22] in 2007, mainly being due to differences in straw yields (t ha^−1^) since crop areas for wheat have marginally increased in the time between the two studies. The current study estimated straw yields on the basis of straw use area and number of straw bales (of particular weights) obtained. By contrast [22], estimated straw yields on the basis of regional crop production areas, harvestable straw yields, and harvest indices (total biomass and grain ratios) from literature, government data and industry representatives. The importance of straw yield variations to national straw supply was highlighted in Denmark where theoretical changes in straw yield could alter the national supply by up to 800,000 tonnes per year (calculated total national supply for Denmark was 5.5 Mt per year) [15]. Our findings in relation to linking straw and grain yields highlight the dangers of estimating national straw supply from grain data in England alone, since large variations in straw yield are seen between regions due to a variety of reasons including cereal varietal choice, use and frequency of plant growth regulators in crop production and height above the ground of the combine ‘header’ at harvest. It is worth noting that yields of wheat straw in particular have been strongly influenced by breeding programmes that have introduced shorter straw genetics (*Rht* alleles) into varieties commonly grown by farmers in Western Europe. Variants of the *Rht* semi-dwarfing allele have the effect of reducing crop height, straw length and susceptibility of the crop to lodging, but have no significant effect on total crop biomass [23]. Most modern varieties of wheat grown on UK farms, such as *JB-Diego* and *Duxford*, contain these dwarfing alleles.

Given the estimated 1.45 Mt of cereal straw that is currently incorporated into the soil from arable farm types, it is instructive to consider the potential bioethanol yield that could be derived from this CPSGB. Assuming a conversion of 333 L of ethanol per tonne of straw (75% of theoretical yield from C5 and C6 sugars, *Pers. comm.* G Tucker; potential yields of greater than 90% have also been reported, dependant upon the pre-treatment process used [36]), this feedstock could generate 482.9 M L of ethanol. Accounting for the lower energy value of ethanol (65.75%) in comparison to gasoline [37], this would represent only approximately 1.5% equivalence of the 20,649 M L of petrol consumed in the UK [38]. However, this biomass source provides an opportunity to achieve this energy substitution without direct land use change leading to a reduction in food or feed supplies. As of 2010, 631 M L of motor spirit (petrol) were derived from bioethanol, representing 2.0% of total petrol consumption energy equivalence. Thus, there is arguably considerable potential for CPSGB to replace or add to this current level of bioethanol demand. However, while first generation biofuel technology is arguably well developed, the derivation of second generation biofuel technology is currently under on-going development supported by industry and government, for example, the BBSRC Sustainable Bioenergy Centre in the UK [11]. Moreover, the estimated 1.5% petrol equivalence noted above is based upon a theoretical potential yield; it is unlikely that it will be economically viable under industrial scale technology to extract a 75% theoretical yield and that lower yield levels can therefore be anticipated. The results in section 3 demonstrate the considerable variation in wheat, barley and oilseed rape production in England together with current straw yields and use across the GORs. It can be argued that the variation in straw yield demonstrates additional potential straw production possibilities; if straw production on arable farm types in the East of England achieved similar levels to the East Midlands, this would generate a further 500 kt of (currently incorporated) wheat straw equating to 167 M L of bioethanol. In addition to CPSGB, DESGB feedstock also offers potential. Current estimates [16] indicate that 3000 and 8000 ha of short rotation coppice and miscanthus are respectively grown in the UK. Given estimates of 14.2 and 5.3 oven dried t ha^−1^ for SRC and miscanthus respectively [39] and assuming conversion of feedstock to ethanol in line with cereal straw conversion, these two dedicated energy crops would generate 85 kt of feedstock and 28.3 M L of ethanol or 0.09% of current petrol consumption. While dedicated energy crops remain an ‘infant industry’, the potential for growth in these crops remains; relatively modest area changes in the production of dedicated energy crops, combined with cereal straw supply could therefore represent a larger contribution towards the road transport fuel obligations target. However, crucially, the conversion of both dedicated energy crops and cereal straw is dependant upon second generation biofuel technologies which are currently under development. Hence, although there is potential for both dedicated energy crop and cereal straw to contribute to the UK's fuel needs, current theoretical estimates of fuel replacement potential is both low and dependant upon emerging technologies. Wider issues of land use impacts are equally important, with the use of feed crops and dedicated energy crops both raising direct and indirect land use change concerns [40]. The potential advantages of the use of cereal straw include the ability to combine both food and fuel production alongside known farm-level production techniques which utilise current on-farm technology.

The legislative requirements to achieve 10% of transport energy from renewable sources by 2020 has thus far largely incentivised the private sector to invest in first generation biofuels, and within the USA legislative changes are now incentivising commercial scale investment in second generation biofuels (USA, Public Law 110-140 (2007)). The results in this paper help to place in context the UK potential for second generation biofuel, particularly that derived from co-products, indicating that cereal straw offers substantial possibilities as a feedstock to either replace or add to the current supply of transport biofuels. Geographically, the majority of English cereal straw that is currently incorporated into the soil is located in the East Midlands and East of England, in line with the major arable areas of the country and where current straw markets are less developed. However, within these arable areas, issues of soil carbon and organic matter depletion prevail [17], albeit often on the basis of anecdotal rather than quantitative analysis, as noted by Ref. [23]. However, Powlson et al. [31] specifically consider issues of soils organic carbon (SOC) from straw removal for bioenergy purposes. They note that SOC was only observed to increase in six out of 25 experiments of cereal straw incorporation, but also cite evidence of detrimental soil health issues associated with reductions in SOC. However, in commercial practise, even where straw is removed, there is a substantial proportion of the straw and stubble biomass that is returned to the soil [30], [31] and this has been argued to explain the relatively small changes in SOC observed from straw incorporation in comparison to continual straw removal [31]. Moreover, an approximate linear relationship was observed between frequency of straw incorporation/removal and relative changes in SOC relative to continual straw incorporation [31]. Hence, these findings arguably support the rotational, in preference to continual, removal of straw given the need to maintain soil health and the benefits of straw as a soil enhancer with respect to nutrient retention [13] and organic matter status [24]. Issues of soil health feature strongly in the current CAP proposals [25]; policy-influences have directly affected farmer decision making in the past and will continue to do so. CAP reform could therefore lead to changes in straw use decisions, particularly in the major arable areas of England, and both the public and private sectors need to consider these policy issues in any further development of the bioenergy sector. It is therefore important that any future market for straw accounts for farmers' attitudes towards using straw in relation to a range of factors, including SOC, soil nutrient status, timeliness of cultivation and crop establishment operations and CAP support mechanisms. One potential further avenue for mitigation of straw removal and soil health concerns, over and above rotational straw removal, could be for lignocellulosic energy plants to return the organic residue from the production process back to farmers supplying cereal straw. However, the chemical and physical properties of the residue would need to be fully evaluated prior to implementation of a ‘straw-supply and residue-return’ policy; the method of feedstock pre-treatment (e.g. mechanical, chemical, biological) will impact upon the properties of the digestate [36] and moreover different configurations of the biorefinery process lead to different uses of process by-products (e.g. for process heat or lignin to generate electricity) [41] which will additionally impact upon the extent of the process residue produced. Nevertheless, cereal straw arguably offers an immediate lignocellulosic feedstock supply without diverting land from food production, with appropriate mechanisms for management of SOC via rotational straw supply and/or residue return policies in place. Given the potential to contribute towards the renewable transport fuel requirements, policy makers may consider following legislators in the USA by embedding specific targets for lignocellulosic fuel within the renewable transport fuel obligation [42] in the EU. The corollary would be for policy makers to reduce targets for renewable fuel originating from first generation feedstocks.

## 5 Conclusion

With the growing debate over food versus fuel generated from first generation biofuels, the utilisation of crop residue co-products offers substantial potential as a sustainable biofuel feedstock. On the basis of an England-wide on-farm survey of arable farmers' use of cereal straw combined with production data from the FBS for England, the results presented suggest that the volume of cereal straw from arable farm types that is currently incorporated in the soil, largely in the East Midlands and East of England, could provide sufficient feedstock to meet 1.5% of the UK's petrol consumption requirements. However, issues in the form of farmers' attitudes towards the use of straw, and in particular the benefits of straw as an enhancer of soil nutrient and physical properties, remain. This paper has presented data on production and current uses of cereal straw; information on farmer attitudes towards cereal straw supply including further barriers and incentives to using straw for biofuel production will be of further interest. Perceptions of the benefits of straw incorporation, and a better understanding of the magnitude of these benefits, are important areas for further research for both policy makers and the fuel industry alike.

## Figures and Tables

**Figure:**
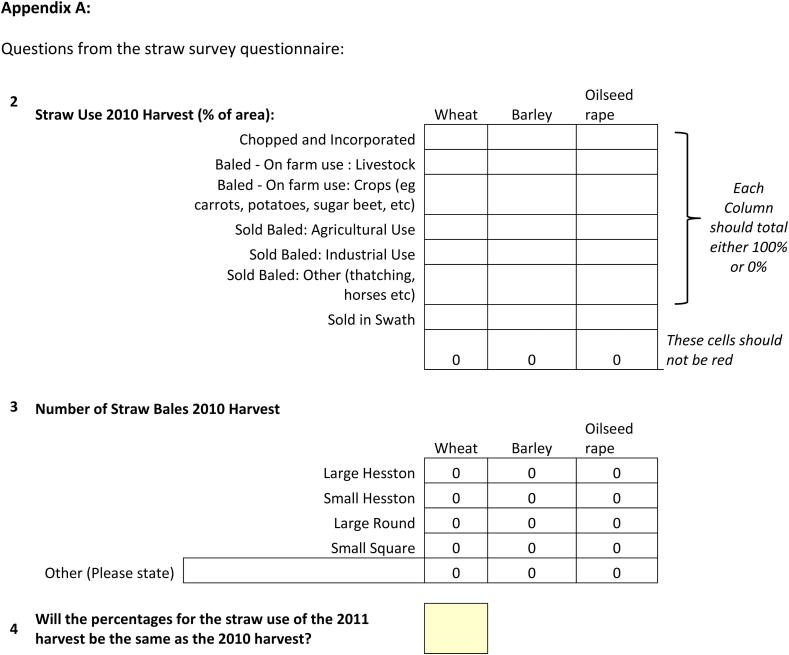


**Table B1:** Bale weights used in the analysis for this study

Large Hesston^a^	500 kg
Small Hesston^a^	240 kg
Large Round^a^	170 kg
Small Square^a^	15 kg
Mid range Hesston^b^	370 kg
Very small bales 10–12 kg/bale^c^	11 kg
Class Quadrant and D1010^b^	245 kg
Large square^d^	170 kg

a Data from http://www.hay-straw-haylage.co.uk/bale-sizes.html (accessed 2/4/12).

b Author calculation based on data from http://www.hay-straw-haylage.co.uk/bale-sizes.html (accessed 2/4/12).

c Value provided by RBR RO.

d Taken to be the same as a large round.

**Table 1:** Observations by farm type and GOR.

GOR	Cereals	General cropping	Mixed
North East	8	1	6
North West	7	5	4
Yorkshire and Humber	13	5	11
East Midlands	31	8	7
West Midlands	5	8	7
East of England	30	24	9
South East	20	3	9
South West	11	4	13

**Table 2:** Crop areas (hectares) on arable farm types by GOR and straw use.

GOR (GOR Ref number)	Crop	Chopped incorporated	Baled for on-farm use livestock	Baled on farm use other	Sold baled agricultural use	Sold baled industry use	Sold baled other	Sold in swath
North East (1)	Wheat	9114	23,181	0	18,075	0	0	11,651
	Barley	0	16,310	0	5001	0	0	10,822
	Oilseed rape	17,930	2253	0	3970	0	0	483
North West (2)	Wheat	0	412	682	6464	0	869	15,639
	Barley	297	6018	0	6640	0	2827	2546
	Oilseed rape	4012	0	0	0	0	0	290
Yorkshire & Humber (3)	Wheat	39,563	45,962	20,948	46,627	6113	1087	59,983
	Barley	0	41,452	771	28,263	0	7386	12,385
	Oilseed rape	55,223	977	1527	0	0	2237	21,757
East Midlands (4)	Wheat	120,161	68,304	8832	28,226	0	10,763	103,773
	Barley	706	19,618	0	15,046	0	1651	22,671
	Oilseed rape	141,465	3382	0	0	0	0	1992
West Midlands (5)	Wheat	39,354	29,213	546	10,073	0	0	68,038
	Barley	0	10,389	0	5777	0	609	18,321
	Oilseed rape	45,183	760	0	0	0	1005	0
East of England (6)	Wheat	311,424	34,254	4951	51,950	0	2002	78,314
	Barley	60,737	23,469	353	10,333	0	6011	17,572
	Oilseed rape	131,871	0	0	2963	0	0	2314
South East (8)	Wheat	64,285	60,246	0	52,205	1858	7888	35,725
	Barley	0	14,518	0	27,075	0	3443	12,216
	Oilseed rape	66,621	9685	0	4337	0	0	0
South West (9)	Wheat	0	36,532	1341	37,249	0	0	61,801
	Barley	0	23,336	0	14,858	0	2480	29,938
	Oilseed rape	34,814	3515	0	7896	0	392	3109
England	Wheat	583,901	298,104	37,300	250,869	7971	22,609	434,924
	Barley	61,740	155,110	1124	112,993	0	24,407	126,471
	Oilseed rape	497,119	20,572	1527	19,166	0	3634	29,945

**Table 3:** Straw yields, uses and potential on arable farm types.

Crop	GOR	Area in GOR	Straw yield (t/ha)	Potential total yield (t)	Yield used (t)	Yield incorporated (t)	Percentage used	Percentage incorporated
Wheat	1	62,021	2.52	156,114	133,172	22,941	85.30	14.70
	2	24,066	2.21	53,093	53,093	0	100.00	0.00
	3	220,285	2.76	606,894	497,895	108,999	82.04	17.96
	4	340,059	3.26	1,108,195	716,611	391,584	64.66	35.34
	5	147,223	1.88	277,353	203,215	74,139	73.27	26.73
	6	482,895	1.66	800,943	284,406	516,537	35.51	64.49
	8	222,206	3.34	741,744	527,155	214,589	71.07	28.93
	9	136,923	2.23	305,467	305,467	0	100.00	0.00
	Total	1,635,678	2.48	4,049,803	2,721,014	1,328,789	67.19	32.81
Barley	1	32,132	2.38	76,475	76,475	0	100.00	0.00
	2	18,328	2.00	36,647	36,053	594	98.38	1.62
	3	90,258	3.04	274,486	274,486	0	100.00	0.00
	4	59,692	3.58	213,753	211,224	2530	98.82	1.18
	5	35,096	1.81	63,449	63,449	0	100.00	0.00
	6	118,475	1.95	230,685	112,422	118,264	48.73	51.27
	8	57,252	2.92	167,090	167,090	0	100.00	0.00
	9	70,611	2.25	158,641	158,641	0	100.00	0.00
	Total	481,845	2.53	1,221,228	1,099,840	121,387	90.06	9.94
**Cereal Total**		2,117,523		5,271,031	3,820,855	1,450,176	72.49	27.51
Oilseed rape	1	24,636	1.49	36,593	9961	26,632	27.22	72.78
	2	4303	1.49	6391	431	5960	6.75	93.25
	3	81,722	1.49	121,384	39,359	82,026	32.42	67.58
	4	146,839	1.49	218,105	7982	210,123	3.66	96.34
	5	46,948	1.49	69,734	2622	67,112	3.76	96.24
	6	137,148	1.49	203,711	7839	195,873	3.85	96.15
	8	80,643	1.49	119,782	20,827	98,955	17.39	82.61
	9	49,726	1.49	73,859	22,149	51,710	29.99	70.01
	Total	571,964	1.49	849,560	111,169	738,390	13.09	86.91

**Table 4:** GOR straw yields Ranked (weighted yields).

Rank	Wheat	Barley
1	South East	East Midlands
2	East Midlands	Yorkshire & Humber
3	Yorkshire & Humber	South East
4	North East	North East
5	South West	South West
6	North West	East of England
7	West Midlands	West Midlands
8	East of England	North West

Key: 1 greatest straw yield; 8 lowest straw yield.

## Footnotes

1 The UK Cereals event describes itself as ‘the leading technical event for the UK arable industry’ with *circa* 27,000 visitors annually.

2 The other type of bale required both the number of bales and the type to be stated.
